# Building blocks for meta-synthesis: data integration tables for summarising, mapping, and synthesising evidence on interventions for communicating with health consumers

**DOI:** 10.1186/1471-2288-9-16

**Published:** 2009-03-04

**Authors:** Rebecca E Ryan, Caroline A Kaufman, Sophie J Hill

**Affiliations:** 1 Cochrane Consumers and Communication Review Group, Australian Institute for Primary Care, La Trobe University 3086, Victoria, Australia; 2 University of Massachusetts Medical School, 55 Lake Avenue North, Worcester, MA, 01604, USA

## Abstract

**Background:**

Systematic reviews have developed into a powerful method for summarising and synthesising evidence. The rise in systematic reviews creates a methodological opportunity and associated challenges and this is seen in the development of overviews, or reviews of systematic reviews. One of these challenges is how to summarise evidence from systematic reviews of complex interventions for inclusion in an overview. Interventions for communicating with and involving consumers in their care are frequently complex. In this article we outline a method for preparing data integration tables to enable review-level synthesis of the evidence on interventions for communication and participation in health.

**Methods and Results:**

Systematic reviews published by the Cochrane Consumers and Communication Review Group were utilised as the basis from which to develop linked steps for data extraction, evidence assessment and synthesis. The resulting output is called a data integration table. Four steps were undertaken in designing the data integration tables: first, relevant information for a comprehensive picture of the characteristics of the review was identified from each review, extracted and summarised. Second, results for the outcomes of the review were assessed and translated to standardised evidence statements. Third, outcomes and evidence statements were mapped into an outcome taxonomy that we developed, using language specific to the field of interventions for communication and participation. Fourth, the implications of the review were assessed after the mapping step clarified the level of evidence available for each intervention.

**Conclusion:**

The data integration tables represent building blocks for constructing overviews of review-level evidence and for the conduct of meta-synthesis. Individually, each table aims to improve the consistency of reporting on the features and effects of interventions for communication and participation; provides a broad assessment of the strength of evidence derived from different methods of analysis; indicates a degree of certainty with results; and reports outcomes and gaps in the evidence in a consistent and coherent way. In addition, individual tables can serve as a valuable tool for accurate dissemination of large amounts of complex information on communication and participation to professionals as well as to members of the public.

## Background

Systematic reviews have developed into a powerful method for summarising and synthesising evidence derived from trials and other predominantly experimental studies, and their use by decision makers in clinical and policy settings is increasingly promoted and accepted [1]. The rise in the number of systematic reviews creates a new methodological opportunity and associated challenges and this is seen in the development of overviews, or reviews of systematic reviews (Ch. 22 [2]). One of these challenges is how to summarise evidence from systematic reviews of complex interventions for inclusion in an overview. Complex interventions are so called because of the dynamic, multifaceted, interacting and socially contingent nature of the interventions and their application [3].

### Interventions for communication and participation

The Cochrane Consumers and Communication Review Group coordinates the production of Cochrane systematic reviews of interventions which affect consumers' interactions with healthcare professionals and the health system. In describing the work of the review group, we use the term 'consumers' to include patients, their family carers, or members of the public with an interest in health. For brevity, we describe the wide range of interventions which affect people's interactions as interventions to communicate with and promote the participation of consumers in health care. Many of the interventions covered by the scope of the review group would be classified as complex according to the UK's Medical Research Council definition mentioned above [3]. A few examples illustrate both the complexity and diversity: interventions to improve consumers' adherence to medicines could include instruction, counselling or psychological therapy, simplified dosing, self-monitoring, reminders, lay health mentoring, crisis or family interventions, and many others, and these can each be delivered alone or as complex multifaceted interventions [4].

Broadly, interventions for improving communication and increasing participation aim to promote consumers who:

• are more knowledgeable and competent;

• are able to express and exchange their views and beliefs,

• make treatment and healthy behaviour choices alone or with health professionals;

• seek or provide support if required;

• minimise risks and harms;

• are able to access high quality information and quality health services; and

• participate in policy making, planning, service improvement and research.

These aims will therefore relate to the wide range of outcomes being sought, and which might affect individuals (consumers or professionals), health services and systems, as well as contribute to societal goals, for example, healthier populations.

As indicated, interventions for communication and participation are characterised by the diversity and multiplicity of their purposes and the complexity of the situations surrounding their use. They may operate at the level of individuals, groups, systems, organisations or society, they may be applied through many different media in addition to human interaction, and their delivery may involve many players. For example, contract-based interventions to improve adherence to treatment, screening or health promotion may be directed at patients, family carers and/or professionals providing these services [5]. Some interventions may be developed to be disease-specific, although many have general application across populations (or could be adapted to be so). Consequently, many reviews coordinated by the Consumers and Communication Review Group are broad in focus, rather than narrow, thus making them relevant and important for the treatment and management of many diseases, and to the formation of health policies for more responsive health systems.

### Overviews of the effects of interventions for communication and participation

The World Health Organization, through its Regional Office for the Western Pacific, has developed a policy framework for a more responsive and people-centred health system [6] and during this process commissioned the Consumers and Communication Review Group to provide summaries of systematic reviews to inform the development of the framework. At the time the project commenced (2004), methodology for the preparation of overviews or reviews of systematic reviews was at an earlier stage. Our view in 2004 was that overviews could play an important role in informing policy, health professionals and consumers about the state of evidence, enabling summary and comparison across a wide range of intervention types and settings. The issue we debated was whether an overview should be a straightforward summary of the results of included systematic reviews, that is, it would be not much more than a listing of summaries of data largely derived from the abstracts of reviews, or alternatively it could provide more information and be in a format that enabled a 'higher' or meta level of analysis.

Decisions on which data to collect and analyse are framed by the question that is posed. In the case of a systematic review, the question might be what are the effects of interventions to improve adherence to medicines [4], whereas an overview might ask the broader question of what interventions directed to consumers improve the use of medicines – including, for example, procurement, consumers' understanding, and medical outcomes as well as adherence [7]. However, in seeking to contribute to the development of policy, and in recognition of some of the challenges of summarising review-level evidence for complex interventions, we opted to provide more than the information that is currently provided in a typical abstract of a review. This choice led us to develop a method that would enable standardised messages on the evidence, its uncertainties and evidence gaps, and which would be concise but sufficiently informative for policy deliberation [8].

The process of summarising systematic reviews of interventions for communication and participation was more difficult than we anticipated and in the course of the work for WHO and other bodies subsequently, we have developed a method of summarising and assessing data from systematic reviews which is also an output. We call the output 'data integration tables'. The term data integration table was chosen because it indicates key inputs to this process: data that are selected, a process of integrating data, and data that are tabulated (either for stand-alone purposes, such as dissemination of the review results, or for inclusion in the next (higher) stage of analysis). The output is therefore both a series of steps and a table: the tables themselves can then be used (entire or modified) in different ways. This article describes the developmental process of the data integration tables. In relation to other key terms, and in common with others [2], we call a review of systematic reviews an overview and we term the method of analysis in an overview meta-synthesis. This article does not describe the analytical process of meta-synthesis but it does consider the implications of the data integration tables in the context of conducting a meta-synthesis.

### Developments in the science of synthesis and meta-synthesis

As mentioned above, the production and use of systematic reviews has risen at pace with the increasing amount of primary literature available. As the science of research synthesis has developed interest has also grown in ways to combine and present review-level evidence in accessible, consistent formats [8-13].

Overviews have been used to respond to clinical questions such as the effectiveness of a range of interventions for a particular condition (Ch. 22 [2]); or the effects of the same intervention across conditions. Cochrane Collaboration review groups, including the Cochrane Musculoskeletal, Cochrane Menstrual Disorders and Subfertility, and the Cochrane HIV/AIDS Groups, have developed methods and prepared overviews to address these types of questions [14-16]. Others have focussed on addressing the needs of policy makers in relation to evidence-based decision making in health priority areas [17]. This work has involved the development of rating schemes to standardise and report outcomes. Using these tools, the strength of evidence derived from reviews can be rated and summarised in relation to key outcomes.

Methodological approaches to preparing overviews vary according to their aims (for instance, to be an input to policy deliberation, to develop specific recommendations, or to inform the field about the effects of different strategies) and key audiences (this may be dependent on who would implement the interventions). They are also determined by the populations, interventions and outcomes evaluated in systematic reviews. For example, the interventions assessed in clinically-focussed reviews are, in general, less complex and diverse than those seen in areas incorporating social complexity or social process, such as communication and participation in health. Reviews of clinical interventions also typically focus on a relatively narrow range of outcomes that are readily measurable in a clearly defined population. This contrasts with the hugely diverse outcomes, assessed in a multitude of ways, that typify systematic reviews of public health and socially complex interventions [10,18,19]. In these latter categories, a narrative report and synthesis of results rather than statistical meta-analysis is more common, due to the heterogeneity of the included studies. Such differences and others mean that meta-synthesis methods must be developed to accommodate methodological complexity but remain able to be tailored to assist the analytic process. In other words, meta-synthesis must be more than just a listing of findings from individual systematic reviews.

We were unable to find a model of synthesising review-level evidence with direct applicability to the area of communicating with and promoting the participation of consumers in health. Models with a clinical or public health focus provided a basis for approaching assessments and rating of the strength of evidence. However, these approaches did not provide a clear basis for standardising the reporting of diverse outcomes spanning clinical or disease areas, one of the most difficult features of the evidence on communication and participation. Our aim was to develop a model of synthesis that might standardise the evidence and so enable broad questions about the effectiveness of interventions for communication and participation to be answered, such as what helps people to make informed decisions about screening? We therefore sought to develop methods that would deal with the particular features of the evidence on communication and participation and provide us with a basis for the preparation of overviews, or meta-synthesis.

This article describes the developmental process, methodological issues, in association with its output or result, so for ease of understanding we have combined the traditional Methods and Results sections into one. The Discussion section describes the uses of the tables, their strengths and limitations, and future work.

## Methods and results

### Data integration tables: Development and production

Four steps were undertaken in designing the data integration tables: first, relevant information for tabulating key characteristics of a review was identified, extracted and summarised. Second, results for the outcomes of the review were assessed and translated to standardised evidence statements. Third, the outcomes and evidence statements were mapped into an outcome taxonomy that we developed, using language specific to the field of interventions for communication and participation. Fourth, the implications of the review were assessed after the mapping step clarified the level of evidence available for each intervention.

For each of these Steps 1–4, we outline the methods we adopted, methodological issues debated, modifications required and the output or result of each step. Tables 1, 2, 3 illustrate key inputs: see Additional file 1 for a worked example of a data integration table for one entire systematic review.

**Table 1:** Major characteristics extracted from reviews published by the Cochrane Consumers and Communication Review Group

Description of main review characteristics and the data extracted from reviews for each characteristic	
**Aim**	Adapted from review objectives.

**Scope **– *Study design*, *Participants*, *Interventions*, *Comparison arms*, *Outcomes*	Adapted from review selection criteria; the number and type of studies and participant numbers included were also extracted.
**Number and types of studies**	
**Number of participants**	

**Analysis**	Indicates whether meta-analysis and/or narrative data analysis was performed.

**Setting**	Country and predominant settings in which included studies were conducted and interventions delivered.

**Recipient**	Adapted from the Consumers and Communication Review Group scope, which groups reviews via direction of the communication processes (*e.g*. to the consumer; from the consumer; between provider and consumer; between consumers), so describing both the primary intervention recipient and the major direction of communication processes.

**Provider**	Who delivered the intervention to the recipient. Also describes important characteristics such as experience or training required to deliver the intervention.

**Format**	The predominant delivery format(s) of the intervention. May also include important characteristics such as frequency, intensity or delivery to individuals or groups.

**Quality**	Quality of included studies: summary based on review authors' criteria used to rate included study quality and authors' assessment of included study quality.
	Quality of the review: based on AMSTAR; includes the overall quality score and summary of criteria that review methods failed to meet.

**Authors' conclusions**	Authors' conclusions added as a composite of points raised in the Discussion and Implications (for practice, for research) sections of the Cochrane review.

**Table 2:** Assessment scheme for consistent reporting of review results

SUMMARY STATEMENT	TRANSLATION CRITERIA
**Sufficient evidence from trials**	Evidence sufficiently certain to support conclusions about the effect of the intervention(s) in relation to a specific outcome(s). This includes evidence of an effect in terms of (i) benefit or (ii) harm.
	Criteria that need to be met:
	• Statistically significant results are considered to represent sufficient evidence to support conclusions; or
	• The numbers of trials/participants included in the analysis for a particular outcome are also considered. For example: meta-analysis yielding a statistically significant pooled result based on a large number of included trials/participants; or narrative data with statistically significant results, such as 12 studies of 14 showing a significant effect of an intervention on a particular outcome.

**Some evidence from trials**	Less conclusive evidence to make a decision about the effects of a particular intervention(s) in relation to a specific outcome(s).
	Criteria that need to be met:
	• A narrative synthesis of results, with the result qualified according to the review findings, *e.g*., 'some evidence (5 studies of 9) reported a positive effect of ....' A rating of 'some evidence' is based on more equivocal results than those rated as 'sufficient evidence'. For example, while 12/14 statistically significant studies would be classed as 'sufficient evidence', 5/9 statistically significant studies would be rated as 'some evidence'; or
	• A rating of 'some evidence' may also be based on a statistically significant result obtained in a small number of trials; or a statistically significant result obtained from trials with a small number of participants.

**Insufficient evidence from trials**	Not enough evidence to support conclusions about the effects of the intervention(s) on the basis of the included studies. This should be interpreted as 'no evidence of effect', rather than 'evidence of no effect'.
	Criteria that need to be met:
	• Statistically non-significant results; or
	• Where the number of trials and/or participants is small, 'insufficient evidence' may reflect lack of power to be able to detect an effect of the intervention; or
	• Where the number of trials and/or participants is large, 'insufficient evidence' may reflect underlying ineffectiveness of the intervention.

**Insufficient evidence in relation to measurement**	Not enough evidence to support conclusions about the effects of the intervention due to a lack of reporting on the specified outcomes.
	Criteria that need to be met:
	• The review elected not to report on a particular outcome(s) despite being reported by included trials; or
	• The review was not able to report on the outcome, as data for the outcome were not reported by included trials.

**Table 3:** Taxonomy of outcomes for communication and participation

Consumer oriented outcomes
Knowledge and understanding
Communication
Patient involvement in care process
Evaluation of care
Support
Skills acquisition
Health status and well being
Health behaviour
Treatment outcomes

**Health care provider oriented outcomes**

Knowledge and understanding
Consultation processes

**Health service delivery oriented outcomes**

Service delivery level
Related to research
Societal or governmental

Source: 'Outcomes of interest to the Cochrane Consumers and Communication Review Group', published at http://www.latrobe.edu.au/cochrane/assets/downloads/Outcomes.pdf

### Step 1: Summarising key characteristics of reviews

The first step involved identifying information from a review that would provide a context for interpreting it and aid an assessment of applicability by policy makers. This step also included two quality assessments – of the review, and of its included trials. Combined, this information summarised the key characteristics of the systematic review.

#### Identifying and extracting data

We looked systematically across all published Cochrane reviews whose preparation was coordinated by the Consumers and Communication Review Group to identify key categories of information needed to provide a context for interpreting review results. The first issue we faced was how much information to extract. Our experience reading and using very brief summaries of other systematic reviews of complex interventions led us to opt for more, rather than less, categories of information, because brevity sometimes hindered understanding – for example, about the content, format or delivery of complex interventions. In addition, our experience of using reviews of interventions for communication and participation in a policy making context had confirmed our belief that there is confusion in the language, purposes and outcomes of many interventions for communication and participation. In other words, there is a lack of an internationally shared language for many of the main interventions in this field. This meant that we opted for a comprehensive set of data categories with the aim of avoiding potential confusion in the use of the data.

We developed a data extraction table based on these categories, piloted it iteratively and adapted it as required. The main methodological issue we identified through this process was quality assessment. We decided, in line with the increasing international recognition of the importance of quality assessment, that it was necessary to provide a quality assessment of the systematic review and also of the included studies, and so added this to later versions. In addition, authors' conclusions were added as a composite from the Discussion and Implications sections of the Cochrane review in later versions as a consistency check against the final stages of preparing the data integration table.

To prepare tables, one investigator (RR) extracted data from reviews. All data were checked for accuracy and completeness by a member of the team who had prepared the originating Cochrane review.

#### Quality assessment: Quality of the review

The quality of the systematic reviews was assessed using a measurement tool for *Assessment of Multiple SysTemAtic Reviews *(AMSTAR) [20] which has been used by others to assess systematic review quality [21]. This tool assesses the quality of the systematic review process against eleven distinct criteria. A review meeting all eleven criteria is regarded as a review of the highest quality.

#### Quality assessment: Quality of the review's included trials

We used the review authors' assessment of the quality of included trials in each systematic review. The rationale was that Cochrane reviews use rigorous methods, including independent study quality assessment by at least two review authors [2]. All reviews also undergo rigorous peer and statistical refereeing prior to publication. We debated using the GRADE approach [22] for rating the quality of included trials, as it was developing alongside the current methods, but decided against this for two reasons: no reviews prepared for the Consumers and Communication Review Group had yet used this approach, and it would require significant additional work (that is, re-analysis of included studies) to complete ourselves. In future this option may become possible as more Cochrane review authors adopt these new methods.

#### Output of Step 1: Key characteristics of reviews

Table 1 identifies the main characteristics of the review for which data would be extracted and informs researchers where they would usually find that data in a Cochrane Systematic Review, or how it would be described. Data items include: review aim; scope, which includes study design, participants, interventions, comparison arms and outcomes; number of studies; types of studies; number of participants; method of analysis (that is, whether or not a meta-analysis was performed); setting; recipient of the intervention, provider of the intervention, and format; quality of the review and of its included studies; and authors' conclusions. The item 'recipient' classifies information relevant to the direction of communication, is adapted from the scope of the Consumers and Communication Review Group, and recognises that communication is a dynamic social process and not unidirectional.

### Step 2: Assessing the review results

#### Developing an evidence assessment scheme

A scheme to assess the results of reviews was developed to allow consistent reporting of findings across reviews. We examined a range of review-level syntheses produced by different groups who were summarising systematic reviews of complex interventions (including the Health Development Agency, the Community Guide and groups within or affiliated with the Cochrane Collaboration [17]) to determine the types of schemes in use and their characteristics. We decided that the main features of the scheme were that it had to be able to:

• represent accurately and consistently results that were statistically pooled, or narratively reported, or combinations of the two;

• differentiate clearly between results that were reasonably certain; those where effects were less certain or uncertain; and those where data were absent.

Our initial scheme included three standardised evidence statements to summarise review results in relation to a specific outcome: sufficient evidence from trials (results could be regarded as sufficiently certain to support conclusions about effects, either beneficial or harmful); insufficient evidence from trials (not enough evidence to support conclusions about the results); and unclear (not enough evidence to support a firm conclusion, or equivocal results). We piloted this scheme on several reviews, developing an evidence assessment for each and all primary and secondary outcomes identified by the authors of the review. This led to modifications. 'Some evidence from trials' was added to differentiate more clearly the instances where results were more equivocal than would be justified by the description 'sufficient evidence', such as for a review with narrative synthesis where both positive and negative results were reported for a given outcome. It was also necessary to differentiate between a lack of evidence due to statistically non-significant results, and cases where there was lack of evidence due to insufficient reporting. We therefore also added the term 'Insufficient evidence in relation to measurement' to identify those cases where there was a lack of reporting of results on relevant outcomes, either at the included study level, or at the level of the review.

#### Output of Step 2: Assessment scheme

The final scheme is provided in Table 2 and for a worked example of Step 2, see Additional file 1. It provides four assessment statements with criteria that need to be met for each statement. Use of the scheme for subsequent project work [7] has indicated that judgement is still required for the distinction between 'sufficient' and 'some' evidence from trials. Overall, Step 2 aims to ensure consistent reporting of review-level findings for specific outcomes, provide a broad assessment of the strength of evidence derived from different methods of analysis, indicate a degree of certainty with the result, and report gaps in the evidence in a consistent way.

### Step 3: Mapping the evidence

#### Structuring the reporting of outcomes using a taxonomy

The Consumers and Communication Review Group's outcome taxonomy provides a framework with which to organise outcomes relevant to communicating with and involving consumers in health care [23]. It provides a comprehensive pool of outcomes from which to identify those relevant to specific interventions affecting people's interactions with health care. It organises outcomes into three major blocks: consumer-, health care provider- and health service delivery-oriented outcomes (see Table 3). Each block is broken down into broad outcome types relevant to the effects of interventions for communication and participation. These broad types have a thematic coherence that is not present the more one breaks down each category, and at the level of the review, many outcomes are often highly specific, thus making synthesis at a meta level very difficult.

The significance of this step is that we hypothesised that policy makers may find it useful to have outcomes in an overview mapped against a comprehensive but broad set of health-related outcomes, in addition to data being reported for the specific primary or secondary outcomes from the originating review. For instance, a question at overview level might be does X lead to improved knowledge and understanding, changes in behaviour and reduced use of health services. Reviews selected for inclusion into an overview addressing this type of question may not report all these outcomes or report them for a narrowly defined and more specific outcome. In a related methodological field (exploratory efforts to synthesise quantitative and qualitative data in a systematic review [24,25]) researchers had indicated the benefits of a structured report using thematically coherent categories to organise diverse findings [26,27]. We would argue that the taxonomy could provide a set of 'coherent categories' and could be used to map the diverse findings of the review as an input to meta-synthesis. Our hypothesis is yet to be tested but we chose to map results in this way.

#### Output of Step 3: Mapping key outcomes

In practice, there are two small steps under the more general 'mapping' step. The first was to analyse the outcomes identified in each systematic review and to map them to the relevant outcome taxonomy classifications. For example, drawing from O'Connor's Cochrane review on decision aids [28], the outcomes *knowledge*, *accuracy of risk perception *and *realism of expectations *would all map onto the outcome taxonomy category of 'consumer knowledge and understanding'.

Following this, the evidence rating scheme described above was used to report the review's results against each identified outcome type. The result of these steps is that the evidence available for each consumer intervention-related outcome can be gleaned by looking at one column of the results table. Step 3 – in combination with data from the other steps – is not the meta-synthesis but a step towards it.

### Step 4: Synthesis

#### Assessing the implications of the review

Based on the results of the outcome mapping and evidence assessment steps, as well as the review authors' discussion and conclusions, the implications of each review were assessed. The major conclusions represented in the data integration tables are two-fold: the table serves to report the conclusions of the original review authors, but also reports the results of the data mapping and evidence assessment performed by the table author. In this way, gaps in evidence or inconsistencies of reporting can be readily identified.

#### Output of Step 4: Building block for meta-synthesis

In Additional file 1, the final Step 4 is worked through in a systematic review of lay-led self-management. This step does not require re-analysis of the review's component data but is a building block to be used for meta-synthesis. The synthesis statements remain consistent with the review authors' work but also build in the results of the data transformation performed in earlier steps.

This last step, and the tabular format in which the results of each step are presented, facilitate further comparison between reviews, that is, between data integration tables. The resulting 'big picture' vantage point can be employed to draft an overview via meta-synthesis that encompasses large amounts of data.

## Discussion

In this article we have outlined a four-step method for preparing data integration tables to enable review-level synthesis of the evidence on interventions for communication and participation in health. The steps allow for the selection and extraction of relevant data from reviews, incorporation of quality assessment of the review's included studies and an assessment of the systematic review itself. The strength of the review-level evidence is assessed using standardised statements, and findings are mapped in their original form to the outcome taxonomy for communication and participation. Implications are drawn in a consistent format based on the evidence assessments and on the review authors' own conclusions.

### Strengths, uses and limitations of this work

The tables aim to improve the relevance and consistency of reporting on the effects of complex interventions; to allow clear messages about the evidence to be drawn from reviews, particularly around issues of certainty; and to enable systematic identification of gaps in the evidence base. The data integration tables are a building block with which to undertake meta-synthesis and construct overview of reviews. A sharp rise in the number of available systematic reviews has seen demand grow for methods to summarise and synthesise review-level evidence [2,8-13,29]. Overviews are one way of meeting these challenges. In addition, the discrete tables can serve as a valuable tool for accurate dissemination of data in individual reviews to professionals as well as to members of the public.

The data integration tables can be employed for various uses, in addition to the development of overviews. We have utilised and evaluated the format to disseminate new review-level evidence via evidence bulletins: electronic newsletters with wide distribution among professionals and lay people that must maximise content in a limited space [30,31]. Another avenue of data dissemination and improving accessibility to evidence on communication and participation is via publicly available internet-based databases, an example of which is *Rx for Change Database *[32]. *Rx for Change *is administered by the Canadian Agency for Drugs and Technologies in Health and includes evidence derived from systematic reviews that deal with prescribing for and use of medicines by professionals and consumers. We utilised selected components of data integration tables to generate review summaries and to populate the consumer section of this database. As part of this work, a Cochrane overview of systematic reviews is in preparation, addressing the question: what improves evidence-based prescribing for, and medicine use by, consumers [7].

The development of the tables provided us with a relatively succinct format, with well-demarcated sections, allowing for location of key information as well as a more easily digestible presentation. Some Cochrane systematic reviews, particularly those with narrative presentation of results, are substantial documents, and trade-offs had to be made between detail and key messages. With this goal in mind, the data integration table format maintains a comprehensive representation of the original review article, in that key descriptive data is maintained. Mapping outcomes specific to the field of communication and participation, and also performing an assessment of the level of evidence available for each intervention, engenders an end product that is more than the addition of characteristics of the review plus results for review-level data. Rather, the table represents an integration of summary and synthesis. The transparency of process that makes it easily replicable is also a valuable component.

The WHO Patient at the Centre project, which involved gathering and synthesising a broad range of literature, also benefited from the use of data integration tables in the policy formulation process [33]. In conducting this project, time and resource constraints made it impossible to utilise fully the step where outcomes are mapped to the Group's taxonomic categories (Step 3) and at that time, there were insufficient published reviews coordinated by the Group to prepare a stand alone overview addressing a specific question. We have applied Step 3 only to reviews coordinated by the Review Group. We believe that this step would aid meta-synthesis but we remain unsure of its value beyond our Group's reviews and would welcome application by others.

One weakness of our method is that the summary evidence statements still require interpretation, and may not always be easy to apply, particularly for the process of evidence assessment in the context of narrative reporting of results in a review. However, we have used the approach to summarise and assess 19 Cochrane reviews in the *Rx for Change Database *of the Canadian Agency for Drugs and Technologies in Health [32]. User testing of this database is anticipated in the future and may help to clarify areas of confusion or ambiguity.

### Future research

The benefit of the comprehensive nature of the data integration tables is that it allows some extrapolation and application of the results to local contexts – although this is not yet an integrated component. Concurrent with the methodological work described here has been the SUPPORT Project [34], with the development of review summaries and an overview of interventions relevant to primary care [35]. This will influence our ongoing work. We may consider adding a step for 'evidence to practice/policy' or translational issues. This could be derived from the review, in conjunction with consideration of local factors, or could be further developed through focus group research with key decision makers. Other concurrent developments that may influence future research and testing of our approach include the GRADE approach, as mentioned, for rating the quality of included studies [36-38], and the development of lay summaries of Cochrane reviews using the Summary of Findings tables [2,39].

### Implications for policy

We developed our approach in response to the particular challenges we faced in relation to socially complex interventions: various methods of analysis in reviews, complex interventions that are poorly described and understood, and lack of awareness of the range of health outcomes in the field of health communication and participation. Mapping the evidence using the assessment scheme and taxonomy clearly highlights relevant outcomes for interventions promoting communication and participation, differentiates between outcomes where there is sufficient evidence upon which to base a decision and less conclusive bases. The latter would then presuppose a greater role for preferences, value judgements or expert opinion subject to local circumstances [40]. Systematic gap analysis – both for evidence and for outcomes – is also facilitated, leading to easier identification of whether less rigorous research or other study designs will be required to address knowledge gaps. Given this context, we hope that they will also enable evaluation of bodies of evidence as a theoretically-related whole, to assist decision makers by producing a complete and concise representation of the available evidence.

Deficiencies in the patient-centredness of care and poor communication with consumers are persistent and pervasive problems in health care and a large range of interventions now exist to promote active participation and patient-centred care [10,41]. These interventions have the potential to prevent and address the problems associated with communication and participation, and the evidence base from which to make decisions about the effectiveness of these interventions continues to grow [42]. We share with others the challenges of summarising reviews of complex interventions and hope that the methods and outputs described may offer a method for developing coherent overviews for policy makers and other users.

## Conclusion

The data integration tables represent a critical step in creating an overview: data extraction and quality assessment, evidence assessment and mapping to produce consistent messages in relation to a comprehensive set of relevant outcomes. The method provides a transparent and flexible tool for the field of consumer communication and participation. Each data integration table includes relevant information on the context and features of the review, interventions, and results. This information is then evaluated and processed in several steps, and the results of this analysis are also included in the table. The final result is a product that can be used on its own as a method of analysing data from a single review, used to develop derivative products, or, in the future, assembled in combination with other data integration tables, with the purpose of preparing overviews of multiple systematic reviews (meta-synthesis) in the area of consumer communication and participation.

The methods described here deal with the complexities of the evidence base of interventions for improving social interactions and attempt to take account of the challenges of mixed analysis methods, complex and diverse interventions, and multiple outcomes reported in different ways.

## Competing interests

All three authors contribute to the work of the Cochrane Collaboration.

## Authors' contributions

All authors have read and approved the final manuscript.

RER developed, piloted and applied the data integration table format and assessment scheme to published reviews coordinated by the Cochrane Consumers and Communication Review Group. She wrote the first draft of the article and contributed to all versions. CAK applied the evidence assessment scheme to published reviews coordinated by the Consumers and Communication Review Group and contributed to drafts. SJH contributed to the development of the outcome taxonomy, and to the development of the data integration table format and assessment scheme. She contributed to all versions of the article.

## Pre-publication history

The pre-publication history for this paper can be accessed here:

http://www.biomedcentral.com/1471-2288/9/16/prepub

## Supplementary Material

Additional file 1 **Table 4**. Data integration table: Worked example of Steps 1–4. The data illustrates a worked example on a Cochrane review of the complex lay-led self-management intervention, using the methods described in the manuscript. Click here for file
