# Supplementary material for: Building blocks for meta-synthesis: data integration tables for summarising, mapping, and synthesising evidence on interventions for communicating with health consumers
Source: BMC Med Res Methodol. 2009 Mar 4;9:16. doi: 10.1186/1471-2288-9-16 (PMC2678150; doi:10.1186/1471-2288-9-16)
Supplement: Additional file 1 — Table 4. Data integration table: Worked example of Steps 1–4. The data illustrates a worked example on a Cochrane review of the complex lay-led self-management intervention, using the methods described in the manuscript. [file 1471-2288-9-16-S1.doc]

**Additional file 1**

File format: DOC

**Title: Table 4. Data integration table: Worked example of Steps 1-4**

Description: The data illustrates a worked example on a Cochrane review of the complex lay-led self-management intervention, using the methods described in the manuscript.

| **Step 1:**  Summarising key characteristics of reviews | | | |
| --- | --- | --- | --- |
| **Review title**  Self-management education programmes by lay leaders for people with chronic conditions  **Authors [and citation]**  Foster G, Taylor SJC, Eldridge SE, Ramsay J, Griffiths CJ [Self-management education programmes by lay leaders for people with chronic conditions. *Cochrane Database of Systematic Reviews* 2007, Issue 4. Art. No.: CD005108. DOI: 10.1002/14651858.CD005108.pub2.]  **Background**  ‘Lay-led (equivalent to ‘peer-led’) self-management programmes are becoming widespread in the attempt to promote self-care for people with chronic conditions’ (see review).  **Description of main features**  **Aim**  To assess the effectiveness of lay-led self-management programmes for people with chronic conditions.  **Scope (selection criteria)**  Study design:  RCT  Participants:   - *Included:* People of any age with established chronic conditions, defined by the Long-Term Medical Conditions Alliance as ‘an illness of prolonged duration that may affect any aspect of that person’s life.’ - *Excluded:* People without established chronic conditions (*e.g.* those at risk for chronic disease); and those having treatment for cancer (radiotherapy, chemotherapy or therapeutic surgery).   Interventions:   - *Included:* Any lay-led self-management education programme targeting people with chronic conditions. Programmes were included if they were structured and their aim was primarily educational, they primarily addressed disease self-management, and the majority of the content was delivered by lay people. Interventions were included if they incorporated elements of peer support, as long as the primary focus was education addressing disease self-management. Interventions incorporating a clinician-led component were also included, as long as the majority of the time was devoted to lay-led activities. Education could be delivered in various formats, including face-to-face education, delivered in groups or individually. Educational interventions delivered via other media, such as by post or electronically (*e.g*. phone or internet) were included where the activities included an iterative process of interaction between the participant and tutor. Interventions could include education for carers and/or family members, as long as the intervention was primarily focussed on the person with the chronic illness. - *Excluded:* Interventions that did not involve structured formal education, such as those providing information (literature) alone without iteration; and those described as self-management education but were not structured education programmes.   Comparison arms:   - Structured lay-led self-management education programmes for chronic conditions versus usual care or no intervention - Structured lay-led self-management education programmes for chronic conditions versus professional-led programmes   Outcomes:   - *Included:* Primary outcomes incorporated four major categories: clinical outcomes and health status (*e.g.* self-rated health status and health-related quality of life, pain, disability, psychological wellbeing); health behaviour (*e.g.* adherence, cognitive symptom management); healthcare use (*e.g.* hospital admissions, doctor visits); and self-efficacy to self-care.   Secondary outcomes included: knowledge of the condition, social roles/ activities and perceived social support, course attendance, communication with physician, costs of delivering the programme and cost effectiveness, effects on carers/ family members, and adverse outcomes.  **Number of studies included:** 17 (1 study is ongoing)  **Types of studies included:** RCT (16), cluster RCT (1)    **Number of participants included:** 7,442  **Meta-analysis performed:** Yes; narrative data also provided where meta-analysis was not possible.  **Review methods:**  Standard Cochrane Collaboration review methods were used, including the following: *a priori* research design provided; extensive searching; selection criteria were specified in advance and applied; list of included and excluded studies provided; quality criteria for assessment of included studies were reported and applied; methods of analysis were reported; conflict of interest stated. All results are reported at 6 months unless otherwise stated.  **Quality:**  *Included studies:* Assessed via a component approach in terms of: method of randomisation; allocation concealment; blinding of outcome assessors and data analysts; baseline comparability of groups; follow-up; intention-to-treat analysis; validation of tools; and other sources of bias. Overall, trials were of variable quality: two studies were rated as high quality, three of intermediate quality, and for the remainder of included studies quality was unclear. Of the included studies, 7/17 reported adequate randomisation methods; 4/17 adequately concealed allocation; 6/17 adequately blinded outcome assessors, but blinding of data analysts was unclear for all studies; all studies demonstrated comparable groups at baseline; follow-up was variable but none reported follow-up of <65% (2/17 studies achieved follow-up of >90%, 8/17 achieved 81 to 90% follow-up, 3/17 achieved 70 to 80% follow-up, 4/17 achieved rates <69%). ITT analysis was dealt with in different ways: 2/17 studies substituted baseline data for missing data; 1/17 imputed missing values by entering data selected from the sample on a rational basis; 9/17 stated use of ITT analysis but only presented data for study completers; 1/17 reported ITT analysis for some data but not all data; 4/17 did not use ITT analysis. Selective reporting of outcomes may predispose this review to reporting bias: only 7/17 studies identified primary outcomes and many outcomes were reported by a small number of, or single, studies. Participant-expectation bias may also exist as participants could not be blinded to interventions group and outcome measures were self-reported. Use of wait-list control groups may also be prone to bias in these situations: however, this was explicitly assessed by authors in sensitivity analyses and no major changes to results were found.  *Review AMSTAR rating (out of possible 11):* 11 – high quality review.  *Comments:* The review methods adequately met all items of the AMSTAR checklist.  **Setting*:*** *Country:* North America (1 study), UK (4), China (1), Australia (1) and the Netherlands (1). *Intervention:* In the majority of studies (13), interventions were delivered in community settings; three studies delivered interventions in primary care and one in a hospital outpatient setting.  **Recipient:** Interventions directed to the consumer.  **Provider:** Interventions were lay-led by definition. All studies compared interventions delivered by lay leaders with usual care. Three studies also assessed the effects of a professionally-led intervention group in a third study arm. All lay leaders were trained, and in three studies at least one of the lay leaders had the same chronic condition as study participants. In one study a physician led 25% of the classes.    **Format:** A number of intervention groups were found, but with similar underlying components: the Arthritis Self-Management Programme (ASMP) (5 studies, recruiting participants with arthritis (4 studies) and osteoarthritis of the knees or hips (1 study)); the Chronic Disease Self-Management Program (CDSMP) or its variation The Expert Patient Programme (EPP) (7 studies, recruiting participants with variable conditions including hypertension, heart disease, chronic lung disease, arthritis, stroke, diabetes, chronic pain, chronic back pain, chronic fatigue, mental health and neurological conditions); other disease-specific lay-led interventions (5 studies, recruiting participants with diabetes (2 studies), HIV infection (1 study), chronic low back pain (1 study) and heart failure (1 study)).  The ASMP and CDSMP/EPP include a structured course of 6 weekly sessions, each of approximately 2.5 hours, led by at least one trained and accredited lay leaders. Session content includes information on goal setting, problem solving, lifestyle changes, symptom management and communication with health professionals. Participants also receive an educational booklet, manual or videotape that covers the course content. For disease-specific interventions, the course varied in structure from 4 to 7 weekly sessions, each approximately 1.5 to 3 hours. A participant also received literature about their condition, and in 3 studies an additional videotape.  **Authors’ Conclusions:** Authors recommend that further well-designed and reported research is needed to evaluate the effects of lay-led self-management education programmes in several areas. In particular, research is needed on a range of standardised outcomes relevant to chronic disease management, including long term effects beyond 6 months. Additional outcomes that should be assessed include biological/clinical markers of disease control; clearly defined measures of healthcare use and self-efficacy; and cost-effectiveness.  Authors recommend that the effects of disease-specific interventions be assessed in rigorous studies; and that intervention components be systematically varied and evaluated to establish the most effective lay-led educational interventions for chronic disease-self-management. This should include assessment of professionally-led intervention components.  Authors also recommend that the effects of lay-led self-management education programmes be assessed in a broader range of participants, specifically including men, children and adolescents, and targeting those with more severe morbidity.  Authors additionally recommend that complementary qualitative research be conducted to explore how participants experience the interventions and how uptake of the interventions might be improved. | | | |
|  | **Step 2:**  Evidence assessment | **Step 3:**  Mapping | **Step 4:**  Synthesis |
| **Intervention** | **Results of review** | **Outcomes** | **Implications** |
| Structured lay-led self-management education programmes versus usual care or no intervention | Primary outcomes:   - **Sufficient evidence from trials:** compared with usual care, lay-led programmes were associated with improvements in both pain (11 trials, 4683 participants) (SMD -0.10, 95% CI -0.17, -0.04) and disability (8 trials, 3491 participants) (SMD -0.15, 95% CI -0.25, -0.05) at 6 month follow-up, but insufficient evidence to decide between lay-led programmes and usual care for either outcome at 12 months. - **Sufficient evidence from trials:** compared with usual care, lay-led programmes were associated with reduced fatigue (7 trials, 3251 participants) (SMD -0.16, 95% CI -0.23, -0.09). - **Sufficient evidence from trials:** compared with usual care, lay-led programmes were associated with improvements in both depression (6 trials, 2613 participants) (SMD -0.16, 95% CI -0.24, -0.07) and anxiety (3 trials, 1573 participants) (SMD -0.14, 95% CI -0.25, -0.04). - **Sufficient evidence from trials:** compared with usual care, lay-led programmes were associated with improvements in health distress (6 trials, 3061 participants) (SMD -0.25, 95% CI -0.0.34, -0.15) and general self-reported health status (6 trials, 3061 participants) (WMD -0.20, 95% CI -0.31, -0.10), although the latter outcome was associated with significant heterogeneity (p<0.01). - **Sufficient evidence from trials:** compared with usual care, lay-led programmes were associated with higher frequency of aerobic exercise (7 trials, 3040 participants) (SMD -0.20, 95% CI -0.27, -0.12) and frequency of practising cognitive symptom management techniques (4 trials, 2628 participants) (WMD -0.55, 95% CI -0.85, -0.26), although there was significant heterogeneity in the latter outcome (p<0.001). - **Sufficient evidence from trials:** compared with usual care, lay-led programmes were associated with improvements in self-efficacy to manage symptoms (10 trials, 3682 participants) (SMD -0.30, 95% CI -0.41, -0.19), although there was significant heterogeneity for this outcome (p=0.01). - **Insufficient evidence from trials:** to decide between lay-led programmes and usual care with respect to psychological wellbeing, health-related quality of life, shortness of breath or clinical measures (HbA1c levels). - **Insufficient evidence from trials:** to decide between lay-led programmes and usual care with respect to healthcare use, including physician/GP visits, number of days or nights in hospital or emergency room visits.   Secondary outcomes:   - **Sufficient evidence from trials:** compared with usual care, lay-led programmes were associated with improved communication with health professionals (7 trials, 3643 participants) (SMD -0.15, 95% CI -0.25, -0.05), although there was significant heterogeneity in this outcome (p=0.05). - **Insufficient evidence from trials:** to decide between lay-led programmes and usual care with respect to knowledge, social support, programme attendance or costs. - **Insufficient evidence in relation to measurement:** no study reported the effects of lay-led programmes, compared with usual care, on outcomes for carers.   Harms and adverse effects:   - **Insufficient evidence in relation to measurement:** authors note that none of the included studies reported adverse outcomes or harms, and that no study reported that complaints had been received. | Consumer oriented outcomes  Knowledge and understanding   - Insufficient evidence from trials of lay-led programmes on knowledge.   Communication   - Sufficient evidence from trials: Lay-led programmes improve communication with professionals.   Patient involvement in care process   - Sufficient evidence from trials: Lay-led programmes improve self-efficacy to manage symptoms.   Evaluation of care   - Insufficient evidence in relation to measurement.   Support   - Insufficient evidence from trials of lay-led programmes on social support.   Skills acquisition   - Sufficient evidence from trials: Lay-led programmes improve communication with professionals. - Sufficient evidence from trials: Lay-led programmes increase frequency of practising cognitive symptom management techniques.   Health status and wellbeing   - Sufficient evidence from trials: Lay-led programmes improve pain, disability and fatigue at 6 months. - Sufficient evidence from trials: Lay-led programmes improve depression, anxiety, health distress, and general self-reported health status. - Insufficient evidence from trials of lay-led programmes on psychological well-being, health-related quality of life, or shortness of breath. - Insufficient evidence from trials of lay-led programmes on physical or psychological health of carers.   Health behaviour   - Sufficient evidence from trials: Lay-led programmes increase frequency of practising cognitive symptom management techniques and aerobic exercise. - Insufficient evidence from trials of lay-led programmes on programme attendance (adherence).   Treatment outcomes   - Sufficient evidence from trials: Lay-led programmes improve pain, disability and fatigue at 6 months. - Sufficient evidence from trials: Lay-led programmes improve depression, anxiety, health distress, and general self-reported health status. - Insufficient evidence from trials of lay-led programmes on psychological well-being, health-related quality of life, or shortness of breath. - Insufficient evidence from trials of lay-led programmes on clinical (HbA1c) measures.   Health care provider oriented outcomes  Knowledge and understanding   - N/A   Consultation processes   - N/A   Health service delivery oriented outcomes  Service delivery level   - Insufficient evidence from trials of lay-led programmes of effects on healthcare use (physician/GP visits, time in hospital or emergency room visits). | What this review shows about lay-led self-management programmes for people with chronic conditions:  Compared with usual care, lay-led self-management interventions in the short term may improve participants’ self-efficacy to manage symptoms, as well as self-rated health and use of cognitive symptom management techniques. They may also reduce health distress.  Compared with usual care, lay-led self-management interventions may in the short term improve measures of pain, disability, fatigue, depression and anxiety; however these effects are small and are not likely to be clinically important.  What this review does not show about lay-led self-management programmes for people with chronic conditions, *i.e.* gaps in the evidence:  Compared with professional-led interventions, the effects of lay-led self-management interventions on health and treatment outcomes, behavioural outcomes, consumer knowledge, involvement and evaluation of care.  Compared with usual care or professionally-led interventions, the effects of lay-led self-management interventions on health service use outcomes (including doctor visits and time in hospital), clinical outcomes, psychological wellbeing overall or health-related quality of life.  The effects of lay-led self-management interventions on consumer-oriented and other outcomes (professional and health services outcomes) beyond 6 months. |
| Structured lay-led self-management education programmes versus professionally-led programmes | Primary outcomes:   - **Some evidence from trials:** compared with a professionally-led programme, a lay-led programme was associated with more frequent practice of relaxation techniques (1 trial, 86 participants).   Other outcomes:   - **Insufficient evidence from trials:** to decide between lay-led and professionally-led interventions with respect to knowledge or locus of control (diabetes).   Harms and adverse effects:   - **Insufficient evidence in relation to measurement:** authors note that none of the included studies reported adverse outcomes or harms, and that no study reported that complaints had been received. | Consumer oriented outcomes  Knowledge and understanding   - Insufficient evidence from trials: of lay-led programme effects on knowledge.   Communication   - Insufficient evidence in relation to measurement.   Patient involvement in care process   - Insufficient evidence from trials: of lay-led programme effects on locus of control. - Insufficient evidence in relation to measurement: effects on self-efficacy.   Evaluation of care   - Insufficient evidence in relation to measurement.   Support   - Insufficient evidence in relation to measurement.   Skills acquisition   - Insufficient evidence in relation to measurement.   Health status and wellbeing   - Insufficient evidence in relation to measurement.   Health behaviour   - Some evidence from trials: professionally-led programs better than lay-led for frequency of relaxation technique practice. - Insufficient evidence in relation to measurement: effects on program attendance, attitudes or health-enhancing lifestyle changes.   Treatment outcomes   - Insufficient evidence in relation to measurement   Health care provider oriented outcomes  Knowledge and understanding   - N/A   Consultation processes   - N/A   Health service delivery oriented outcomes  Service delivery level   - Insufficient evidence in relation to measurement: effects on costs. - Insufficient evidence in relation to measurement: effects on health services utilisation. - Insufficient evidence in relation to measurement: effects on adverse events and harms.   Related to research   - N/A   Societal or governmental   - N/A |
